# Rapid Assessment Procedure Informed Clinical Ethnography (RAPICE) in Pragmatic Clinical Trials of Mental Health Services Implementation: Methods and Applied Case Study

**DOI:** 10.1007/s10488-018-0909-3

**Published:** 2019-03

**Authors:** Lawrence A. Palinkas, Douglas Zatzick

**Affiliations:** 1 Department of Children, Youth and Families, Suzanne Dworak-Peck School of Social Work, University of Southern California, 669 W. 34th Street, Los Angeles, CA 90089-0411, USA; 2 Department of Psychiatry and Behavioral Sciences, University of Washington, Seattle, WA, USA

**Keywords:** Pragmatic clinical trials, Implementation, Qualitative methods, Clinical ethnography, Policy

## Abstract

Pragmatic clinical trials of mental health services are increasingly being developed to establish comparative effectiveness, influence sustainable implementation, and address real world policy decisions. However, use of time and resource intensive qualitative methods in pragmatic trials may be inconsistent with the aims of efficiency and cost minimization. This paper introduces a qualitative method known as Rapid Assessment Procedure-Informed Clinical Ethnography (RAPICE) that combines the techniques of Rapid Assessment Procedures with clinical ethnography. A case study is presented to illustrate how RAPICE can be used to efficiently understand pragmatic trial implementation processes and associated real world policy implications.

## Introduction

Pragmatic clinical trials (PCTs) have come to play an increasingly important role in mental health services research (Brierley et al. 2013; Aakhus et al. 2014; Priebe et al. 2015; Thomas et al. 2015; Wenborn et al. 2016). A PCT compares treatments under everyday clinical conditions and are designed to directly support real world policy decisions regarding the use of an intervention (Califf and Sugarman 2015; Loudon et al. 2015). The design is summarized by the pragmatic-explanatory continuum indicator summary (PRECIS) (Thorpe et al. 2009; Loudon et al. 2015). Pragmatic trial study participants are more representative than those recruited in traditional explanatory RCTs because eligibility criteria are often less strict. The PCT is also designed so data can be easily collected in clinical settings under conditions of everyday practice. Pragmatic trials can include questions from and important to multiple diverse stakeholders, including policy makers. PCTs therefore have the potential benefit of producing research that is actionable by being designed around application to practice, with an emphasis on successful implementation (Tunis et al. 2003; Glasgow et al. 2005). Other key pragmatic trial domains include intent-to-treat data analytic approaches, incorporation of research procedures that minimize the need for adjudication (i.e., reaching consensus), and the efficient rollout of PCTs in real-world health care systems. The National Institute of Health Common Fund has supported the NIH Healthcare System Research Collaboratory in an attempt to catalyze methods development in the design and rollout of pragmatic clinical trials (https://www.nihcollaboratory.org/Pages/default.aspx).

A key aspect of pragmatic trials is an emphasis on efficiency in research processes so as to minimize costs per subject randomized (Zatzick et al. 2016). In part, this efficiency is achieved through the use of hybrid effectiveness-implementation designs in which assessments of program effectiveness and implementation outcomes may be evaluated simultaneously. There are three types of hybrid designs (Curran et al. 2012). Type I designs are primarily focused on evaluating the effectiveness of the intervention in a real-world setting, while assessing implementation is secondary; Type II designs give equal priority to an evaluation of intervention effectiveness and implementation; and Type III designs are primarily focused on the evaluation of an implementation strategy and, as a secondary priority, may evaluate intervention effectiveness, especially when intervention outcomes may be linked to implementation outcomes. Each design makes use of both quantitative and qualitative methods in mixed methods designs. In Hybrid I designs, for instance, quantitative methods are typically used to evaluate intervention or program effectiveness, while mixed methods are used to identify potential implementation barriers and facilitators. Mixed methods are used in Hybrid 2 designs to evaluate both process and outcomes of program effectiveness and implementation. In Hybrid 3 design, while quantitative methods are typically used to evaluate effectiveness, mixed methods are used to evaluate both process and outcomes of specific implementation strategies (Palinkas and Cooper 2018).

Although several pragmatic clinical trials have incorporated the use of qualitative methods in their designs (Brierley et al. 2013; Littlewood et al. 2015; Orgeta et al. 2015; Thomas et al. 2015; Wenborn et al. 2016), such methods are often both time and labor intensive (Green et al. 2015), and, thus, are inconsistent with the aims of PCTs. Moreover, the designs of these studies are not based on PRECIS standards (Loudon et al. 2015). In the absence of established guidelines for use of qualitative methods in PCTs (Glasgow and Riley 2013), our understanding of implementation processes in PCTs required for sustainable service delivery is limited (Palinkas and Cooper 2018; Zatzick et al. 2016).

Over the past decade, members of the investigative team have established a stakeholder partnership with the American College of Surgeons (ACS) Committee on Trauma (College) whereby the results of behavioral health pragmatic clinical trials can be translated into policy requirements and best practice guidelines for the regulation of US trauma care systems nationally (American College of Surgeons Committee on Trauma 2006, 2014; Love and Zatzick 2014; Terrell et al. 2008; Zatzick et al. 2016). The ACS oversees the development of national policy requirements and clinical best practice guidelines that inform the integrated operation of US trauma centers and affiliated trauma care systems (American College of Surgeons Committee on Trauma 2006, 2014). The ACS has successfully linked trauma center funding to verification site visits and other quality indicators (American College of Surgeons Committee on Trauma 2006, 2014).

In this paper, we introduce Rapid Assessment Procedure-Informed Clinical Ethnography (RAPICE), an approach to collection and utilization of qualitative data in pragmatic clinical trials of mental health services implementation that is informed by clinical ethnography and rapid assessment procedures. Additionally, an illustrative case study is presented that highlights how RAPICE can help understand the interplay between pragmatic trial behavioral health implementation processes and associated trauma center policy.

## Clinical Ethnography

Ethnography refers to both a process and outcome of research that produces an interpretation and description of a particular group, organization, or system, including behaviors at all levels, customs, norms, roles, and methods of interaction (Bernard 1988; Green et al. 2015). It is typically carried out through participant observation and in-depth interviews, with the researcher immersing him/herself in the regular, daily activities of the people involved in the setting. In most cases, this is a long-term investment of time and energy, with regular observation occurring over weeks, months or years.

Participant observation is the “process of learning through exposure to or involvement in the day-to-day routine activities of participants in the research setting” (Schensul et al. 1999, p. 91). “It involves establishing rapport in a new community; learning to act so that people go about doing their business as usual when you show up; and removing yourself every day from cultural immersion so you can intellectualize what you’ve learned, put it into perspective, and write about it convincingly” (Bernard 1988, p. 148). Participant observation can take many forms, ranging from exclusive participation to exclusive observation (Geertz 1976; Rosaldo 1984; Luhrman 1989; Zatzick and Johnson 1997).

Clinical ethnography is defined as culturally- and clinically-informed self-reflective immersion in local worlds of suffering, healing, and wellbeing to produce data that is of clinical as well as anthropological value. This approach seeks to combine and balance the anthropological method of participant-observation with clinical evaluation of others and reflexive evaluation of self (Calabrese 2013). It is a method involving an immersive ethnographic study of illness and clinical practice, with the additional intent and result of improving clinical outcomes (Kleinman and Benson 2006; Lie et al. 2011). A key feature of clinical ethnography is the training of clinicians in ethnographic methods (Brody 1981; Dao et al. 2018).

There are numerous examples of the application of ethnographic methods in clinical settings, including mental health settings. Ware and colleagues (1999), for example, used ethnographic methods of field observation and open-ended interviewing to investigate the meaning of continuity of care in mental health services. Observations were carried out at two community mental health centers and a psychiatric emergency evaluation unit in Boston. Sixteen recipients and 16 services providers at these sites were interviewed. In a follow-up study, the investigators used ethnographic data to develop (Ware, Dickey et al. 2003) and validate (Ware, Tugenberg et al. 2003) the trustworthiness of CONNECT, a measure of continuity of care. Willging and colleagues used ethnographic methods and a review of legal documents and state monitoring data to examine the impact of Medicaid reform on mental health services (Willging et al. 2005) and the transfer of all publically funded behavioral health services under the management of one private corporation in New Mexico (Willging et al. 2009).

In dissemination and implementation (D&I) research, participant observation and ethnography are commonly paired with in-depth interviews and quantitative data collection to produce a more comprehensive understanding of the ways in which a project was implemented, describe the extent of fidelity to the intervention, and identify and understand barriers and facilitators of implementation (Torrey et al. 2012). Researchers often use key informant interviews, in-depth interviews as well as group interviews, to create a detailed account of the implementation process and its context (Green et al. 2015). For instance, Brunette and colleagues (2008) examined the extent to which an integrated dual disorders intervention was implemented by 11 community mental health centers participating in a large study of practice implementation. Trained implementation monitors conducted regular site visits over two years, interviewing key informants, conducting ethnographic observations of implementation efforts, and assessing fidelity to the practice model. These data were coded and used as a basis for detailed site reports summarizing implementation processes. Palinkas and colleagues (2008) used ethnographic methods of participant observation and extended semi-structured interviews with trainers, clinical supervisors, and clinicians representing 11 agencies in Honolulu HI and Boston MA to understand predictors and process of treatment implementation in the Clinic Treatment Project (CTP), a randomized effectiveness trial of evidence-based treatments for depression, anxiety, and conduct problems in children.

## Rapid Assessment Procedures

While clinical ethnography seems to be an approach that is well suited to mental health services research, it is usually applied in a manner similar to classic ethnographic techniques, which are used over a long period of time, usually by a single individual. One method for efficient collecting and analyzing qualitative data in a relatively shorter period of time is a technique developed by anthropologists known as Rapid Assessment Procedures or RAP. This approach is designed to provide depth to the understanding of the event and its community context that is critical to the development and implementation of more quantitative (cross-sectional or longitudinal) approaches involving the use of survey questionnaires and diagnostic instruments (Scrimshaw and Hurtado 1987). Distinguishing features of RAP include: (1) formation of a multidisciplinary research team including a member or members of the affected community; (2) development of materials to train community members; (3) use of several data collection methods (e.g., informal interviews, newspaper accounts, agency reports, statistics) to verify information through triangulation; (4) iterative data collection and analysis to facilitate continuous adjustment of the research question and methods to answer that question; and (5) rapid completion of the project, usually in 4–6 weeks (Beebe 1995; Harris et al. 1997). Essentially, the methodology is based on techniques of participant observation and non-directed interviewing.

Rapid assessment procedures have been used in evaluation studies of healthcare organization and delivery (Vindrola-Padros and Vindrola-Padros 2018). For instance, it has been used to assess the mental health service needs of older adults (Palinkas et al. 2007) and victims of school shootings (Palinkas et al. 2004); to provide information on values, beliefs and cultural perspectives necessary for designing effective and socially valid health education programs, such as the design of a HIV/AIDS prevention program in East Los Angeles based on how Latinas view virus transmission (Scrimshaw et al. 1991); and to develop materials relevant to health promotion and disease control, such as posters depicting Oral Rehydration Salts replenishing fluids lost from bouts of diarrhea and vomiting (Herman and Bentley 1992). It has been used to conduct formative program evaluations to make midcourse corrections to improve program effectiveness, such as UNICEF’s efforts to provide leaders with information on parts of a program that needed improvement (Pearson 1989); and to design summative program evaluations, such as the assessment of effects of primary health care programs on nutritional and health practices of families (Scrimshaw and Hurtado 1987). It has been used to generate hypotheses to be tested in large scale studies, such as the collection and analysis of focus group data to determine factors influencing decision of parents to have children immunized (Coreil et al. 1989); and to collect information on topics that are time-dependent, such as providing the UN with information needed to determine how to prioritize funding for Primary Health Care (PHC) programs (Scrimshaw and Hurtado 1987).

RAP has also been used in conducting evaluations of program implementation (Goepp et al. 2008; Schwitters et al. 2015; Wright et al. 2015). For instance, Ackerman and colleagues (2017) used “rapid ethnography” to understand efforts to implement secure websites (patient portals) in “safety net” health care systems that provide services for low-income populations. Site visits at four California safety net health systems included interviews with clinicians and executives, informal focus groups with front-line staff, observations of patient portal sign-up procedures and clinic work, review of marketing materials and portal use data, and a brief survey.

## Rapid Assessment Procedure Informed Clinical Ethnography (RAPICE)

The procedures that constitute the RAPICE method are outlined below. The RAPICE method is initially presented with the potential for application to a broad range of pragmatic trial contexts. This description of RAPICE methods is then followed by a specific case example of the use of RAPICE in a pragmatics trial in the acute care medical setting.

In 2011, Zatzick and colleagues articulated the combination of RAP and clinical ethnographic techniques in developing and implementing a single site stepped collaborative care comparative effectiveness trial targeting PTSD and related comorbidities (Zatzick et al. 2011). The method combined intensive participant observation by the ethnographically informed clinician with periodic review of field observations with the clinically oriented social scientist. As with RAP, RAPICE applied in a pragmatic clinical trial is distinguished by the following: (1) formation of a multidisciplinary research team including a member or members with clinical and/or administrative expertise and ethnographic and mixed methods training, enabling efficiency in data collection and analysis through division of labor; (2) development of materials to train team members in ethnographic methods and rapid assessment procedures that minimize the burden placed on any single study participant; (3) use of several data collection methods (e.g., participant observation, informal and semi-structured interviews, field jottings and logs, quantitative surveys) to verify information through triangulation; (4) iterative data collection and analysis in real time to facilitate continuous adjustment of the research question and methods to answer that question; and (5) rapid completion of the mixed method component of the project, which may vary depending on project aims and mixed method design.

When combined with quantitative methods in mixed method designs, RAPICE can be used to collect and analyze data to address important research questions that govern the principles and practice of implementation science. These include the following: (1) what factors act as barriers and facilitators to implementing a specific evidence-based policy, program or practice in a specific setting or context; (2) do these barriers and facilitators exist in other settings or contexts; (3) what are the characteristic features of the process of successful implementation; (4) what strategies are associated with successful implementation of specific policies, programs and practices; (5) how do we determine whether or not these strategies are successful; and (6) why are these strategies effective in producing successful implementation outcomes?

Procedures for the collection and analysis of ethnographic data on implementation context, process, barriers, facilitators and outcomes are outlined as a series of steps in Table 1. These steps can be further condensed into three stages. The first stage involves field observations by the clinical team of participant observers and a periodic review of that data with the mixed methods consultant. This stage usually begins with preliminary telephone discussions with potential sites, followed by training calls and training site visits. The training site visits are often conducted by the study Principal Investigator who, along with other members of the research team, acts in the capacity of a participant observer (PO). During the visit, the PO participates in meetings with site staff, collects whatever documents are available that record procedures implemented, and completes field notes and jottings that are audio recorded and later transcribed by research study staff in order to log site visit and trial activities. Informal and semi-structured interviews with site staff participants can occur before, during, or after the training site visits. The second stage involves pragmatic data analysis that includes discussion and coding of the qualitative data by team members. During this stage, a formative evaluation of the intervention may be conducted to assess the need to adapt the intervention for the specific setting and population and identify requirements for specific adaptations. The third stage involves translation of the data analysis to inform suitability of the intervention for a particular population or service delivery context, new intervention designs and methods for testing in subsequent clinical trials, and evaluation of the factors contributing to the outcomes of the current implementation trial.

The approach to documentation of site visit and trial activities simultaneously aims to satisfy the pragmatic trial requirements for the minimization of time and resource intensive research methods that require extensive adjudication (Thorpe et al. 2009; Loudon et al. 2015) and the implementation science goal of understanding and documentation of trial processes that could yield sustainable maintenance of screening and intervention procedures (Curran et al. 2012; Zatzick et al. 2016). In the case study described below, logs and field notes were subsequently reviewed on an approximately monthly basis with the investigation’s mixed methods consultant (MMC); however, such reviews can be conducted more frequently (e.g., weekly) depending on project aims and requirements. The MMC reviews the data and then queries the PO to gain more insight into the data and its context. These queries then provide a framework for a preliminary interpretation of the meaning and significance of data by the PO, expressed in terms of a set of a priori and emergent themes and their interrelationship. The MMC then provides his/her own interpretation of the meaning and significance of the data using the same format of a priori and emergent themes and interrelationships. A discussion ensues until both the PO and the MMC reach consensus as to the meaning and significance of the data. RAPICE data collection may be conducted by masters-level investigators with appropriate training in conducting ethnographic fieldwork and semi-structured interviews. Review of collected data and preliminary analyses with a doctoral level researcher with expertise in mixed methods is recommended.

Data analysis in RAPICE can incorporate different styles of analysis that vary markedly in resource and adjudication intensity. The immersion/crystallization style can be used exclusively in scenarios where pragmatic trial resources devoted to qualitative analyses are minimal. The immersion/crystallization style is used to provide interpretations of the data by both the PO and MMC, and “consists of a prolonged immersion into and experience with the text and then emerging, after concerned reflection, with an intuitive crystallization of the text” (Miller and Crabtree 1992, p. 19). In this analytic approach, the investigators prepare short descriptive statements or “memos” to document initial impressions of topics and themes and their relationships (Miles and Huberman 1994).

The more time intensive editing style utilizes a methodology of focused thematic analysis (Saldana 2016) that would be appropriate to better-resourced mixed method pragmatic trials. For focused thematic analysis, field notes documents and interview transcripts are independently coded by the PO and MMC to condense the data into analyzable units. Segments of text ranging from a phrase to several paragraphs are assigned codes based on a priori (e.g., from a semi-structured interview guide) or emergent themes (also known as open coding, Strauss and Corbin 1998). Following the open coding, codes are assigned to describe connections between categories and between categories and subcategories (also known as axial coding, Strauss and Corbin 1998). Lists of codes developed by each PO are matched and integrated into a single codebook, constructed through a consensus of team members and consisting of a list of themes, issues, accounts of behaviors, and opinions that relate to organizational and system characteristics that influence program or practice implementation. Based on these codes, computer programs like QSR NVivo or Atlas.ti may be used for well-resourced pragmatic studies to generate a series of categories arranged in a treelike structure connecting text segments grouped into separate categories of codes or “nodes.” These nodes and trees are used to further the process of axial or pattern coding to examine the association between different a priori and emergent categories. They are also used in selective coding of material to identify the existence of new, previously unrecognized categories, and specific examples of co-occurrence illustrated with transcript texts. Through the process of constantly comparing these categories with each other, the different categories are condensed into broad themes using a format that places implementation within the framework of the organizational and system characteristics.

As with thematic analysis, template analytic techniques can be introduced into better-resourced pragmatic trial designs. “Template analytic techniques all share the use of a template or analysis guide, which is applied to the text being analyzed” (Miller and Crabtree 1992, p. 19). The template can be used to generate themes, patterns and interrelationships and is based on theory, research tradition, pre-existing knowledge, and/or a summary reading of the data that occurs when using the immersion/crystallization analytical style. Templates can be used to identify the presence or absence of a priori barriers and facilitators to implementation using constructs from established frameworks like the CFIR, to make comparisons of the presence or absence or characteristics of themes elicited from data collected at sites or from different categories of participants (e.g., clinicians versus patients or administrators), to document changes in occurrence of themes over time, or to integrate quantitative and qualitative data.

Because RAPICE is conducted in clinical settings, reporting requirements and assessment of quality should be consistent with standards that have been proposed for health services that emphasize rigor (Cohen and Crabtree 2008; O’Brien et al. 2014). One of the best illustrations of this approach is the Consolidated Criteria for Reporting Qualitative Studies (COREQ; Tong et al. 2007), a 32 item instrument that assesses quality in three domains: research team and reflexivity, study design, and analysis and findings. However, because of the role of participant observation and reliance on the immersion/crystallization style of data analysis, it may also be appropriate to adopt the standards proposed by Charmaz (2014) that is consistent with constructivist grounded theory and lists 19 criteria grouped under the categories of credibility, originality, resonance, and usefulness.

## Applied Case Study

### Design Overview

Over the past decade, members of the investigative team have developed and rolled out two large-scale effectiveness-implementation hybrid pragmatic clinical trials in the acute care medical practice context, the Disseminating Organizational Screening and Brief Intervention (DO-SBIS) and the Trauma Survivors Outcomes and Support (TSOS) investigations. Both trials simultaneously aimed to impact clinical effectiveness for patient outcomes while also targeting national trauma center implementation policies. Conducted between 2007 and 2012, DO-SBIS randomized twenty US level I trauma centers to usual care control (n = 10) and intervention (n = 10) conditions (patient N = 878) (Zatzick et al. 2013, 2014). An ACS policy summit was scheduled in the final years of the trial to facilitate sustainable implementation of results derived from trial effectiveness findings. TSOS began in 2014 and is ongoing (Zatzick et al. 2016). The TSOS trial aims to enhance the implementation of evidence-based screening and interventions for PTSD and comorbidity for 25 level Level I trauma centers nationwide. As with the DO-SBIS trial, an ACS policy summit is scheduled in the final years of the trial in order to enhance the direct translation of study research findings into national trauma center regulatory policy. The TSOS study began with a small pilot investigation conducted at two trauma centers in the Western United States. Institutional review board approval was obtained prior to the implementation of both the DO-SBIS and TSOS pragmatic trial research protocols. Also, informed consent was obtained from the individual provider participants included in both studies prior to the conduct of semi-structured interviews.

### Participant Observation; Field Notes, Jottings, and Logs

Previously articulated procedures for the documentation of clinical trial activities were adapted for the current pragmatic trial approach (Palinkas et al. 2004; Zatzick et al. 2011, 2016). Study team members recorded field notes and jottings using a standardized format outlined by Bernard (1988). Logs were also compiled, completed and edited during and after study team orchestrated ACS policy summits. These logs were combined into summaries of policy discussions and reviewed as part of the RAPICE data analyses in order to identify and categorize varying degrees of policy activity.

### Provider Key Informant Interviews

Over the course of the DO-SBIS and TSOS investigations, study team members conducted semi-structured key informant interviews with consenting front-line providers to better understand the interplay between the rollout of study procedures and national ACS policy requirements and best practice guidelines. Interview data used in this case study derive from the end-of-study DO-SBIS provider interviews (Zatzick et al. 2014), as well as interviews conducted as part of the TSOS pilot investigation (Zatzick et al. 2016). Using a semi-structured interview guide, providers were asked to discuss experiences with the rollout of alcohol screening and brief intervention services required by the ACS. Providers were asked to describe the nature of services implemented as well as barriers to and facilitators of the implementation of these services. In the TSOS pilot, providers were also asked to compare the mandated rollout of alcohol screening and brief intervention services with the guideline-recommended but not required implementation of PTSD screening and intervention services. The provider interviews were audio-recorded and transcribed verbatim by research assistants.

### National Trauma Center Behavioral Health Surveys

In an effort to triangulate data derived from other RAPICE sources, results from a national trauma center survey conducted by the study team were used to augment the results of the qualitative analyses. Specifically, survey results were used to substantiate, on a national level, individual participant reports of policy dictated uptake of behavioral health screening and intervention services. The study team conducted a survey of all US Level I trauma centers between June 2006 and November of 2007 to assess alcohol screening and brief intervention practices before the widespread institution of College alcohol policy requirements (Terrell et al. 2008). Trauma centers were asked if they routinely screened for alcohol and whether or not they delivered evidence-informed brief alcohol interventions to patients with alcohol use problems. The study team conducted a second survey between August of 2011 and July of 2012, and again asked Level I trauma center sites about alcohol screening and brief intervention practices, as well as trauma center procedures for PTSD (Love and Zatzick 2014).

### RAPICE Case Study Data Analyses

In this case study, RAPICE was used to better understand how various levels of ACS policy facilitated, constructed barriers to, or failed to address key acute care medical implementation processes associated with a pragmatic trial rollout. The RAPICE method allowed the study team to review multiple data sources including field notes and jottings, semi-structured interview data, policy summit logs and national trauma center practice surveys. The spectrum of ACS policy activity and initiatives was assessed using a RAPICE-informed review of logs derived from prior study team sponsored ACS policy summits. Pragmatic trial implementation processes were described using the Reach, Effectiveness, Adoption, Implementation and Maintenance (RE-AIM) framework, a widely adopted set of measures of implementation outcomes (Glasgow et al. 1999; Forman et al. 2017; Glasgow and Chambers 2012; Zatzick et al. 2016). The template analytic style of data analysis was used to integrate quantitative and qualitative outcome measures.

Limited resource allocations for mixed method assessments in the DO-SBIS and TSOS studies necessitated a pragmatic trial data analytic framework that emphasized time efficiency and minimal adjudication; the immersion/crystallization analytic approach was used to provide interpretations of the data. Due to pragmatic trial requirements for conducting analyses in a time-efficient manner, the PO often reviewed data in real time and presented monthly summaries to the MMC, rather than undertaking extensive, time intensive coding procedures.

The RE-AIM framework was used to develop categories of pragmatic trial implementation processes that could be informed by the multiple data sources collected by the study team. For example, national survey data informed observations of the reach of behavioral health screening and intervention, while semi-structured interview data facilitated more granular assessments of the implementation and maintenance of high quality behavioral health screening and intervention procedures. As study team members iteratively reviewed data from these multiple sources, a series of themes emerged related to the interrelationships between ACS regulatory policy and site level implementation processes. Study team logs of policy summit proceedings identified four categories of ACS policy activity including: (1) policy requirements, (2) clinical practice best guideline recommendations, (3) description of an activity without a requirement or guideline, and (4) no mention.

## Results

An example of the data collected from semi-structured interviews conducted during the site visits is provided in Supplemental Data Table 1. The interview transcript was obtained from a staff member at one of the sites participating in the DO-SBIS study. Analysis of this transcript by the MMC suggested two possible overarching themes: use of non-evidence based practice alcohol interventions and sustainment of non-evidence based practice alcohol interventions. The first theme addressed the RE-AIM Adoption outcome and included procedures for conducting an assessment of current alcohol use and a psychoeducational intervention. The assessment included questions regarding how alcohol is used, changes in drinking behavior, reasons for use the day of injury, and opinions about safety and effects of drinking. The psychoeducational intervention included review of drinking hazards, evaluation of pros and cons of drinking, plans for seeking help, establishment of goals, and sources of support. The second theme addressed the RE-AIM Maintenance outcome and focused on sustainment of the alcohol assessment and psychoeducational intervention and included plans for staffing and identification of barriers to sustainment (i.e., limited time and interest). Initial analysis by the PO revealed some slightly different themes, as well as some convergent coding. Initially, themes related to training (Adoption) and implementation of alcohol screening and brief intervention were discussed. Next, the content of the intervention delivered during the trial was explored. Finally, the impact of the study termination and staff turnover was discussed with regard to the maintenance of high quality alcohol screening and intervention going forward. The semi-structured interview revealed that the prior pragmatic trial had built upon existing resources to provide an alcohol screening and brief intervention program that adequately addressed ACS screening and brief intervention verification criteria. Additional observations regarding the trauma center’s alcohol screening and brief intervention implementation were made by the PO in the years after DO-SBIS during the conduct of TSOS. Field observations revealed that site staff turnover had continued and that additional training would be needed in order for the site to regain the higher quality alcohol screening and brief intervention that had been delivered during DO-SBIS.

### Alcohol Screening and Brief Intervention Field Jotting

Three themes derived from consensus coding procedures were observed in a field jotting related to a TSOS trauma center site visit (Supplemental Data Table 2). The first theme involved interactions with trauma center staff present at the site visit; these discussions revolved around trauma team roles in recruitment and intervention delivery. A second set of themes related to team discussions of injured patient cases enrolled. These elements of the field jotting described patient and family characteristics. The final set of themes related to the site’s approach to maintenance of College regulatory requirements targeting alcohol screening and brief intervention. While the provider conducting the alcohol screening and brief intervention performed a mandatory consult, evidence-based motivational interviewing brief intervention approaches were not implemented.

During piloting of the TSOS trial, providers were asked to reflect on the progression of ACS behavioral health policy beginning with alcohol and extending to PTSD. Consensus coding identified multiple themes in this interview segment. The first theme pertained to familiarity with the ACS guideline prior to the pragmatic trial. In this instance, the study participant was not aware of the screening protocol recommended by the guideline: “actually, the first time I heard about it was when you contacted us and started talking about PTSD, and then I did go to the orange book and look at what the college [ACS], what their recommendation was.” The second theme pertained to prior experience with PTSD screening. At this site, staff had conducted PTSD screening, albeit with a protocol that was not outlined in the ACS guideline. According to the provider, “we have been doing a screening for PTSD in our trauma follow-up clinic for many years. We had a questionnaire that the patients would score and then those that scored at a certain level, we would try to get into some type of treatment.” Finally, a third theme pertained to current practice in screening for PTSD. When asked if the PTSD screening was still being used, the provider commented on the inability to screen all patients for PTSD (Reach):

It still would be happening, unfortunately we don’t administer it to every patient, which I think from this study that we’ve done, the incidence of PTSD is so much greater than we probably expected. And so, when the surgeons or the mid-levels see a patient and they feel like perhaps PTSD is a problem for them, then they would ask for the screening to be done. And I think, going forward, it should be done on everybody.

### Logging of ACS Policy Summit Activity

Supplementary Data Table 3 presents a distillation of information logged as part of an ACS Committee of Trauma Policy Summit orchestrated by the study team in September of 2016. The distillation provided a general overview of summit activities while also articulating specific objectives and deliverables. Analysis of the log identified three categories of ACS policy activity, requirement, guideline level recommendation, and suggested mention but no current verification specifications. The log also listed disorders for which ACS requirements (Alcohol), guideline level recommendations (PTSD), and suggested mention but no current specifications (patient-centered care) exist. In summary, while alcohol screening and intervention had been required by the ACS, and PTSD screening, intervention and referral recommended, verbiage about patient-centered care has been recommended for discussion in the resources guide without a recommended verification plan or action.

### National Trauma Center Survey Data on Alcohol Screening and Brief Intervention Uptake

As evidence of the Reach of the intervention, the two trauma center surveys attained 73% (148/204) and 78% (172/221) national level I trauma center response rates respectively. The surveys also documented that prior to the alcohol screening and brief intervention requirement 72.4% of level I trauma centers routinely screened patients for alcohol, and that after the requirement 91.9% of trauma centers screened patients for alcohol (χ(1) = 21.1, P < 0.001). Similarly, before the requirement, 40.7% of centers reported delivering evidence-informed interventions; after the requirement 64.0% of centers delivered an evidence-informed intervention (χ(1) = 17.1, P < 0.001). The second survey also asked questions about routine PTSD screening and intervention; less that 10% of sites reported routine PTSD screening and intervention when the survey was conducted (i.e., 2011–2012).

### ACS Policy and Site Implementation Process Matrix

Table 2 presents the RAPICE-informed integration of pragmatic trial implementation process data as characterized by the RE-AIM framework with categories of ACS policy activity. The matrix derives from a number of data sources including field notes and jottings, semi-structured interviews, national trauma surveys and iterative multidisciplinary team discussions. National trauma center survey data documented the enhanced reach of behavioral health screening and intervention procedures over time. Effectiveness data derived from study team and other pragmatic trials informed ACS policy (American College of Surgeons Committee on Trauma 2006, 2014).

Themes derived from study team discussions were how different types of ACS regulatory policy (i.e., alcohol requirements, PTSD practice guidelines, patient-centered informational additions to the ACS Resource Guide) influenced site-specific adoption, implementation and maintenance processes. With regard to adoption, the study team observed how the alcohol policy mandate required that all trauma centers implement alcohol screening and intervention procedures; trauma centers were required to demonstrate alcohol screening and intervention procedure implementation during ACS verification site visits that occurred every three years. In contrast, clinical practice guidelines and other Resource Guide informational suggestions were only likely to be rolled out by early adopter centers. Over the course of the DO-SBIS and TSOS trials, the study team made longitudinal field observations regarding the extent to which high quality behavioral health screening and intervention procedures were being implemented in the wake of policy requirement or clinical best practice guideline recommendations. Multiple factors, including provider team compositions, trauma center administrative support, staff turnover, and other trauma center resource availability, were observed to influence the longer-term implementation and maintenance of behavioral health screening and intervention procedures. RAPICE-informed study team reviews suggested that while a regulatory mandate successfully required site adoption of alcohol screening and brief intervention, the requirement did not ensure high quality procedures would be implemented and maintained with adequate staffing and resources.

## Discussion

This paper provides an example of the RAPICE-informed integration of implementation science and pragmatic trial approaches as applied to U.S. trauma center behavioral health policy. Over the past decade, members of the investigative team spent hundreds of hours immersed in the acute care medical trauma center clinical context as clinician-investigator participant observers. The study team collected multiple types of data over this period, including field notes and jottings during trauma center site visits and logs of national trauma center policy summit activities. Informal and semi-structured interviews were conducted with trauma center providers involved in pragmatic trial behavioral health screening and intervention procedure rollouts. The study team also conducted two national surveys of trauma center behavioral health screening and intervention uptake over this period. RAPICE allowed for an ideal mixed method for the integration of these multiple data sources and was feasibly embedded within the study team nationwide pragmatic trial rollouts.

As noted earlier, qualitative methods have become a standard feature of implementation research through their use in mixed methods and hybrid effectiveness-implementation designs (Palinkas 2014; Palinkas et al. 2011; Green et al. 2015). However, the use of qualitative methods in PCTs possess a number of challenges, not the least of which is minimizing the cost per subject randomized without sacrificing methodological rigor. There have been several attempts to establish standardized criteria for rigor in qualitative methods. These criteria often have counterparts in criteria used for quantitative methods; hence internal validity is framed in terms of “credibility,” external validity is framed as “transferability,” reliability is framed as “auditability,” and objectivity is framed as “confirmability” (Lincoln and Guba 1985). Although the use of such criteria and the specific elements listed has been a subject of debate among methodologists (Patton 2002; Padgett 2017), they are supported by several strategies designed to enhance rigor and trustworthiness of qualitative methods. These strategies include prolonged engagement, triangulation, peer debriefing and support, member checking, negative case analysis, and auditing (Patton 2002; Padgett 2017; Levitt et al. 2018).

In the DO-SBIS and TSOS, four of these strategies (triangulation, peer debriefing and support, and member checking, and prolonged engagement) were embedded within the RAPICE methodology. Triangulation involved the use of multiple theories or perspectives to interpret the data (theory triangulation), use of participant observation and semi-structured interviews to study implementation process and barriers (methodological triangulation), use of more than one observer during field visits to achieve inter-subjective agreement (observer triangulation), and use of interviews, observational field jottings, and national trauma center survey materials) (data triangulation) (Patton 2002). Further, comparisons of qualitative and quantitative data on similar topics such as organizational culture and climate were conducted to identify points of convergence in a mixed methods design. For instance, as was found in a previous study (Zatzick et al. 2014), there was a lack of congruence between measures of organizational culture and climate (Glisson et al. 2008) that reflected relative stability in trauma centers and ethnographic observations that suggested otherwise. Peer debriefing was conducted between team POs and the MMC. Member checking was conducted through soliciting feedback on preliminary results from participants at an ACS Policy Summit. Finally, the principal investigator has spent hundreds of hours over the past two decades immersed in the acute care medical trauma center clinical context. “The clinician investigator’s in-depth immersive participation in conjunction with collaborative team activity and expert consultant review exemplifies the criteria of prolonged engagement and persistent observation that constitute crucial elements of quality and verification in qualitative research” (Zatzick et al. 2011, p. 125). With regard to prolonged engagement, RAPICE enables time efficient field observation and review procedures that constitute ideal “nimble” mixed method approaches for the pragmatic trial. It also allows for real-time workflow integration.

In addition to the four strategies used in the TSOS study, RAPICE can easily incorporate other strategies for enhancing rigor and trustworthiness such as negative case analysis and auditing. However, as a front-line provider immersed in the clinical setting, the PO’s presentations to the MMC are vulnerable to internal data reduction and selective recall biases. Compared to traditional methods of ethnographic research and semi-structured interviews, RAPICE may be associated with less detailed field notes, fewer observations of important events or implementation processes, fewer and shorter semi-structured interviews with study participants, and less depth in coding, analysis and interpretation of qualitative data collected. These limitations, in turn, may adversely impact the utility of qualitative data in mixed methods designs to achieve the aims of convergence, complementarity, expansion, development, and sampling (Palinkas et al. 2011).

On the other hand, use of RAPICE allows for more opportunities to conduct “repeated measures” of qualitative data through multiple site visits. This represents a different form of prolonged engagement in that the observations may be of shorter duration, but over a longer period of time than is the case with ethnographic methods used in other mental health services research (Ware et al. 1999; Willging et al. 2009). RAPICE also offers richer ethnographic contextual material when compared to implementation research that relies on semi-structural interviews or focus groups because it is able to place the data collected in context in real time. When combined with longitudinal study team field observations in the TSOS Study, RAPICE allowed for an in-depth appreciation of contextual factors that were observed to influence the rollout and sustainability of behavioral health screening and intervention in the wake of ACS policy.

Perhaps most importantly, RAPICE directly addressed pragmatic trial efficiency standards. Rather than requiring time and resource intensive in-depth qualitative research procedures, RAPICE facilitated efficient mixed method data collection and analyses that are in harmony with the pragmatic trial aim of reducing costs per subject randomized through combined input and iterative discussion from both PO and MMC team member perspectives, structuring of roles of research team members to capitalize on their expertise. RAPICE also allows an increasingly more research intensive analytic approach that can potentially incorporate immersion/crystallization, editing/thematic content, and template analysis methods in phases.

The findings of the case study also highlight the importance of understanding the interplay between national behavioral health policy and implementation processes as described by frameworks such as RE-AIM. National trauma center surveys conducted over the past decade documented the enhanced reach of alcohol screening and brief intervention procedures in the wake of ACS policy requirements. Combined with longitudinal field observations, semi-structured interview data highlighted site struggles with staff turnover and the maintenance of high quality screening and intervention procedures over time. Analysis of field jotting data suggested that although alcohol screening and intervention procedures might satisfy ACS verification site visit criteria and thus require adoption by all trauma center sites, the procedures did not adhere to principles derived from the evidence-based practice literature (Zatzick et al. 2014).

Another strength of the RAPICE method is the iterative generation of novel data about implementation processes. The summary matrix derived from field observations, semi-structured interview coding, national survey data and, most importantly, multidisciplinary team discussions, yields important insight into the limitations of implementation strategies that rely on policy requirements alone (Fixsen et al. 2005). While clinical practice guidelines familiarize providers with mental health disorders, and policy mandates make implementation a requirement, the delivery of high quality services and maintenance of adequate staffing and resources are by no means guaranteed, unless further incorporated into ACS policy.

The RAPICE method also may reveal ways in which emerging implementation science constructs may not fully capture novel health service delivery system contextual factors. From an implementation science perspective, acute care medical trauma center inpatient settings are distinct from other primary and acute care settings in that the ACS has the ability to mandate screening and intervention procedures. Thus, the integration of behavioral health screening and intervention services at United States trauma centers is occurring in a unique regulatory “make it happen” context, in contrast to a negotiated “help it happen” implementation context in other general medical settings (Greenhalgh et al. 2004). The ACS has demonstrated its commitment to using empirical data to further the integration of substance use screening and brief intervention at trauma centers (American College of Surgeons Committee on Trauma 2006, 2014).

The description of effectiveness-implementation hybrid trials has not routinely included the targeting of health care policy change as a potential implementation mechanism (Curran et al. 2012). A review of the literature revealed few discussions of the use of mixed methods to better understand the targeted use of health policy as an implementation strategy in effectiveness-implementation hybrid designs (Purtle et al. 2018). The DO-SBIS and TSOS trials may therefore defy categorization as hybrid studies until the novel “make it happen” trauma care systems health service delivery context is better articulated through methods such as RAPICE. The two trials described in the current paper may constitute effectiveness-implementation hybrid designs that use a novel, yet time-tested, ACS policy mechanism as a targeted implementation strategy. The TSOS trial simultaneously aims to determine the effectiveness of the stepped collaborative care intervention model in reducing PTSD symptoms and comorbid conditions, while also assessing the potential utility of the implementation strategy that uses ACS policy to target regulatory mandates for trauma care systems nationally.

RAPICE also provides qualitative data and analysis comparable to that utilized in mixed methods designs in implementation research. In addition to the use of qualitative data for the purpose of convergence described earlier, data collected and analyzed through RAPICE procedures can be used to complement the functions of quantitative methods or expand and explain the findings acquired through the use of quantitative methods (Palinkas 2014; Palinkas et al. 2011).

Finally, RAPICE offers greater transparency in the integration of investigator and study participant perspectives on the phenomena of interest. Unlike Rapid Assessment Procedures as they have been used in public health, the use of RAPICE in this case study did not involve the participation or the insight of community members, or in this case, clinicians participating in the pragmatic trial. Consistent with the aims of the pragmatic trial, input from clinicians at the field sites preceded the implementation of the TSOS. However, in assessing the process of implementation and identification of potential barriers and facilitators, the insider perspective on implementation normally provided by community stakeholders in RAP studies was provided largely by the PO and other study team members (e.g., trauma surgical champions) who simultaneously occupy the dual roles of clinician-investigators and front-line trauma center providers. The outsider perspective was provided largely by the MMC. Consistent with the RAP methodology (Beebe 1995), the two perspectives were integrated in the final analysis through their exchange between the PO and the MMC during the peer-debriefing stage.

## Future Directions

**Fig. 1:**
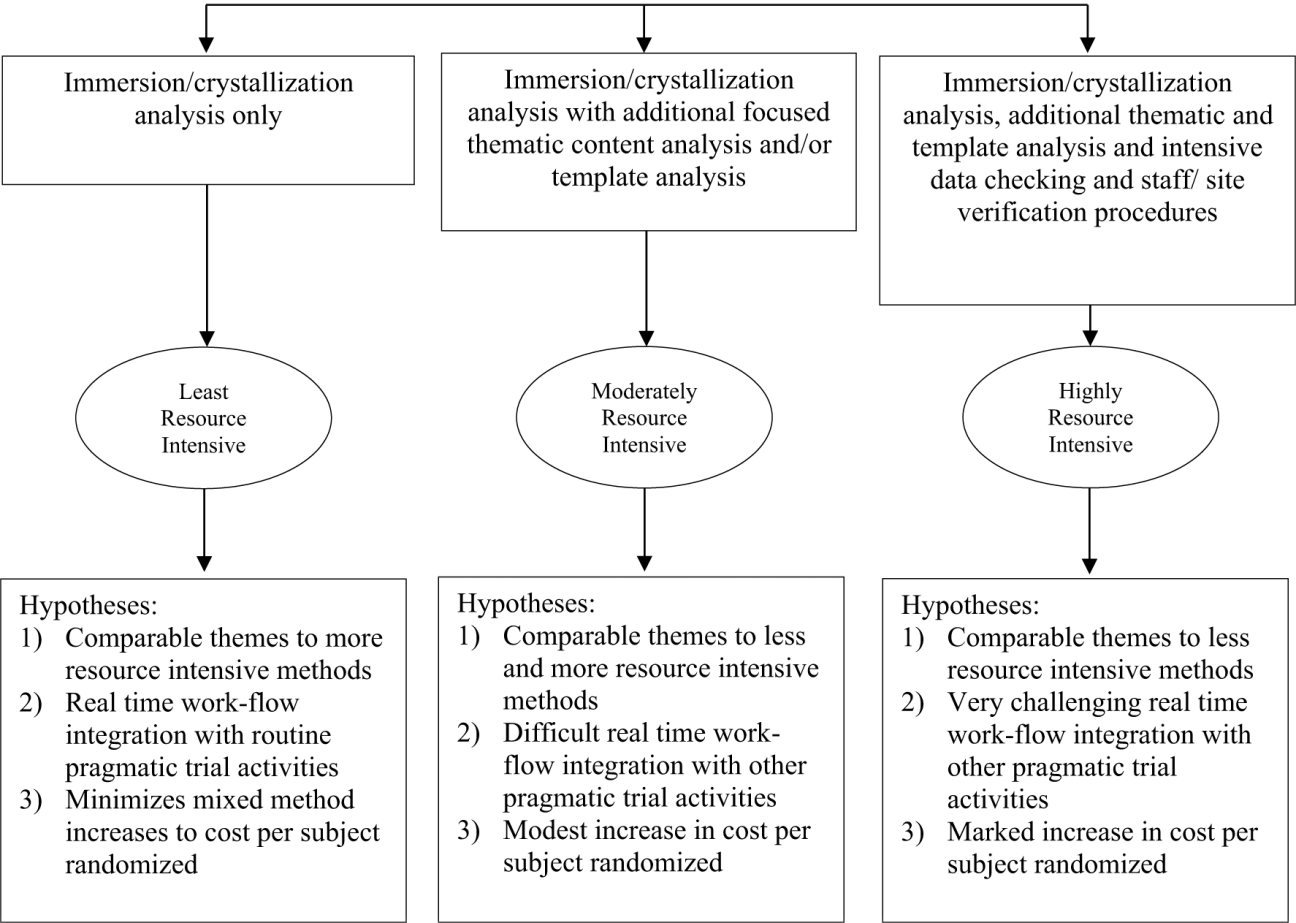
Comparative assessment of three potential RAPICE data analytic approaches with increasing Pragmatic Trial Resource Allocations

Although the use of RAPICE in the TSOS suggested potential in addressing important research questions related to the implementation of evidence-based mental health services in a PCT format, additional research is required to conduct a formal comparison of this methodological approach to standard qualitative methods in implementation research. Such an evaluation might compare RAPICE with more resource-intensive mixed methods on measures such as use of specific strategies to enhance rigor, timeliness, use of research in policy or practice, and cost-effectiveness. For instance, compared to more resource-intensive methods, we hypothesize that RAPICE would provide the same or similar levels of theoretical saturation (Padgett 2017) of information on effectiveness and implementation, but in significantly less time at reduced cost (represented in terms of hours devoted by POs in data collection). Compared with semi-structured interviews and/or focus groups with study participants, we hypothesize that RAPICE would provide the same or greater levels of theoretical saturation (due to potential for triangulating less-structured interview data with data obtained from participant observation) with significantly less burden on study participants (assessed in terms of participant contact time) and significantly reduced cost (assessed in terms of expense to pay staff for conducting such interviews or focus groups and compensations for participants). Such comparisons would enable researchers, policy makers and practitioners to determine whether RAPICE can provide information within the context of a PCT that is similar to that provided by traditional qualitative methods in RCTs, as well as what methodological approach would best meet their needs. Comparisons of different forms of RAPICE that involve increasing degrees of resource and time intensity could be conducted in a similar manner (Fig. 1).

## Conclusions

When applied to evidence-based practice implementation in pragmatic trials, RAP-informed clinical ethnographic methods combine efficient documentation of implementation context, processes and outcomes with periodic review by an expert mixed-method consultant. The methods straddle the continuum between time intensive qualitative procedures and cursory study team review of field observations. Although we have focused here on the potential use of RAPICE in pragmatic clinical trials to examine implementation barriers, facilitators, processes and strategies within specific settings and contexts, we should note that it could be an equally useful in other forms of health research including needs assessments and efficacy and effectiveness clinical trials. Future comparative mixed method investigations could contrast themes derived from RAPICE with themes derived from more in-depth qualitative procedures. In this way, the value added to the clinical trial and implementation processes evaluations by more in-depth qualitative research procedures could be more directly assessed.

In this applied example, the RAPICE approach simultaneously satisfied the pragmatic trial requisite for minimization of time intensive research methods that require extensive adjudication, and the implementation science goal of understanding and documentation of trial processes that could yield sustainable maintenance of screening and intervention procedures through ACS policy. The RAPICE approach furthers the goal of integrating pragmatic trial and implementation science methods by embedding a multidisciplinary implementation team within a health care system pragmatic trial rollout.

## Supplementary Material

Palinkas & Zatzick Supplementary data tables

## Figures and Tables

**Table 1:** Steps to RAP informed clinical ethnography

Stage 1 Participant observer(s) (PO) conduct(s) informal interviews with and/or site visits to participating pragmatic trial organizations PO talks with a spectrum of study participants and visits staff meetings, observes clinical procedures, and/or conducts informal and semi-structured interviews with participants PO writes field jottings of observations and informal interviews, maintains logs of data collection activities and schedules, and digitally records semi-structured interviews for transcription PO presents field notes, logs, interview transcriptions, and any supporting material collected during the site visits to the Mixed Methods Consultant (MMC) MMC reviews the presented data and queries the PO to gain more insight into the data and its context. Suggestions for additional data collection may be provided by the MMC based on assessment of quality and lack of evidence of theoretical saturation
Stage 2 Depending on the pragmatic trial context (e.g., trial resources devoted to mixed methods analyses) qualitative data is subjected to immer-sion/crystalization, and/or focused thematic content analysis, and template analysis PO provides a preliminary interpretation of the meaning and significance of the data, which is organized as set of a priori and emergent themes and description of their interrelationships MMC then provides a preliminary interpretation of the meaning and significance of the data organized in the same fashion and attempts to triangulate findings from multiple data sources (observations, interviews, documents) PO and MMC then identify points of convergence and divergence in the two interpretations A discussion between PO and MMC then ensues until consensus is reached regarding the meaning and significance of the data Depending on pragmatic trial context (e.g., trial resources devoted to mixed methods analyses), if consensus is not achieved, PO conducts follow-up interviews and/or returns to the field sites to collect additional data that could be used to resolve interpretation divergence Depending on pragmatic trial context (e.g., trial resources devoted to mixed methods analyses), if consensus is achieved, MMC makes recommendation for identification of disconfirming cases. PO returns to field sites to collect information that could be used to construct such cases Depending on pragmatic trial context (e.g., trial resources devoted to mixed methods analyses), interpretation of study findings is presented to study participants to confirm validity and comprehensiveness
Stage 3 Analysis of qualitative data collected using RAPICE is then integrated with quantitative data to provide comprehensive understanding of implementation process and outcomes The understandings gained from methods integration is then applied to improve implementation outcomes (formative evaluation) or to address the key study questions related to implementation (summative evaluation)

**Table 2:** American College of Surgeons policy and implementation process summary matrix

Implementation process	Alcohol screening and intervention requirement	PTSD screening and intervention/referral practice guideline	Possible patient-centered care informational section addition to guideline	No mandate or guideline
Reach	Implied that screening should be available for all patients; unclear whether verification site visits check on this or if this data is available within trauma registries	Not addressed	Not addressed	Not addressed
Eiffectiveness	Pragmatic trial literature including DO-SBIS study supports	Pragmatic trial literature supports	Some initial clinical trials, but insufficient for requirement or practice guideline not addressed	Insufficient literature Not addressed
Adoption likely Adopter Groups	Early, Middle & Late Adopters required	Early Adopters likely to uptake	Early Adopters could explore uptake	No adoption likely
Adoption Centers familiar with topic (e.g., alcohol, PTSD)	Mandate enforces familiarity with issue	Guideline suggests familiarity with issue	Informational section introduces/disseminates patient-centered care	Familiarity optional
Implementation Centers required to implement procedure	Mandate requires; verification site visit confirms	Guideline only suggests appropriate practice, no implementation required	Informational section only introduces idea; no implementation required	No requirements
Implementation & Maintenance Adequate staffing and resources allocated	Not addressed; mandate does allow on-site providers to lobby institution for additional staffing and resources	Not addressed; Practice guideline does allow on-site providers to lobby institution for additional staffing and resources	Not addressed; Informational section does allow on-site providers to lobby institution for additional staffing and resources	No requirements
Implementation & Maintenance: High quality procedures implemented and maintained	Not addressed unless specifically required by mandate; on-site providers may be aware of issues related to the variable quality of screening and intervention procedures delivered	Not addressed; on-site providers may be aware of issues related to the variable quality of screening and intervention procedures delivered	Not addressed; on-site providers may be aware of issues related to quality	No requirements
