# Supplementary material for: Rapid Assessment Procedure Informed Clinical Ethnography (RAPICE) in Pragmatic Clinical Trials of Mental Health Services Implementation: Methods and Applied Case Study
Source: Adm Policy Ment Health. Author manuscript; Available in PMC 2020 Aug 14. (PMC7427407; doi:10.1007/s10488-018-0909-3)
Supplement: Palinkas & Zatzick Supplementary data tables [file NIHMS1616145-supplement-Palinkas___Zatzick_Supplementary_data_tables.docx]

**Supplementary Data Tables**

| **Table 1.** Example of interview transcript |
| --- |
| **Research Clinician**: Okay which is totally fine, we basically, I mean the alcohol, the mandate sort of state that you have to have a mechanism to screen for alcohol and then you have to have a potentially an intervention available, but on the control side you were just sort of doing mandate as usual, which is fine, but you know over and above the mandate it sounds like you actually spend some time talking to people. Which is great for your patients and how, what did you, not having been trained in MI, like what were the nature of those interventions? What would you do for thirty to forty five minutes?  **Provider**: Um we would talk about how they used alcohol, if it had increased or decreased, why they had been drinking the day that they had their injury, how they felt about it, how they thought it affected their whole health system, if they thought their drinking was safe or not. We would review then you know how many drinks are too much and how it could affect you. We went over pros and cons of their drinking, we asked stuff like what their family or friends thought of their drinking. If it was, you know, was in the like severe category, then we would talk about you know, do you think you need extra help? How do you get this help? You know what are you gonna do when you achieve your goals? And if you have any goals, and we would talk about goals that they were gonna achieve and how they were gonna reward themselves with these goals at the end to keep on the program. Who they were gonna call as a contact person if they needed to. It was pretty in depth  **Research Clinician:** Okay well, that's fantastic and you know being a control site you know we'll be looking at, I mean that sounds like a pretty good bed side interview and interventions. What's happening now that the study is over? Is that continuing? Or you know what's happening for you now?  **Provider:** Well we have one PA that still left that's doing most of our interventions but not in the depth that Provider S did.  **Research Clinician:** I see.  **Provider**: And then we have a new nurse practitioner that is gonna start soon, that's also been trained in SBI so she's gonna start taking over some of that.  **Research Clinician:** So you still have people that are doing it but they might not be doing it in the same depth that you were doing during the study. Is that mostly a time constraint? Or what's sort of motivating that?  **Provider:** Uh, time and interest. |

| **Table 2.** Field jotting excerpt of alcohol screening and brief intervention example |
| --- |
| Clinician Investigator 1 was met by Provider 1 the trauma nurse taking the lead on the training at 7:45AM. Provider 2, the social work consultation service lead was also at the training as were 2 trauma nurses including Provider 3, and Provider 4. Also at the beginning of the training, were 2 more providers who disciplines included psychology, and nursing, Provider 5, and Provider 6, along with Provider 1. Provider 1 stayed for most of the day as did Providers 2- 4.  The first Trauma Surgeon, Trauma Surgeon 1 worked through an initial case and couple additional cases. The first case involved a recent gunshot victim who was treated at the hospital and had family and friends in need of care. The second case presented was that of an injured homeless patient. The team discussed the cases and went through the full training, and by the end of the day it was decided that Providers 1 and 2 would pick up cases and have weekly staffing rounds.  A second Social Worker came at the end and discussed her alcohol screening and brief intervention. Her screening and brief intervention consisted of a mandatory consult. If the patient is unconscious she might not do anything, and she is not extensively trained in the motivational interviewing brief intervention approach. She is however asking quantity and frequency questions and making referrals for those who show motivation for change. Provider 2 will make an attempt to link up with these consulting services during the intervention |

| **Table 3. American College of Surgeons September 2016 policy summit log** |
| --- |
| The TSOS investigative team is currently conducting comparative effectiveness trials funded by the Patient-Centered Outcomes Research Institute (PCORI) and the National Institute of Health; these comparative effectiveness trials target the delivery of patient-centered and psychosocial/psychiatric care at US trauma centers. The overarching goal of the September 23, 2016 summit is to inform the delivery of high-quality patient-centered and psychiatric care at US trauma center care. The summit would again bring together key stakeholders including key American College of Surgeons’ Committee on Trauma Members, TSOS study investigators, other content experts, and PCORI and NIH program staff. The TSOS study team will be charged with preparing research overviews for each topic presented (e.g., comparative effectiveness trials of patient-centered care at trauma centers, reviews of PTSD, depression, suicide, alcohol and drug screening and intervention at US trauma centers). The overarching goal of the oral presentations and written materials would be to provide the College with information that could inform future updates to the American College of Surgeons’ Resource Guide with regard to patient-centered care and psychosocial/psychiatric care.  **Objectives**  1) Bring together a broad group of stakeholders that include trauma surgeons, mental health professionals, patients, peer advocates, PCORI and NIH staff, and other individuals to discuss research and policy relevant to the delivery of patient-centered and related psychosocial/psychiatric care at US trauma centers  2) Present cutting edge comparative effectiveness studies of patient-centered care delivery in US trauma care systems  3) Review the current literature, practices and American College of Surgeons’ Resource Guide recommendations for screening and intervention for PTSD, depression, suicidal ideation, and alcohol and drug use problems.  **Deliverables**  1) American College of Surgeons’ Resource Guide recommendations regarding patient-centered care for US trauma care systems.  2) Additional recommendations for stress response symptoms, and screening and intervention guidelines for PTSD, depression and associated suicidal ideation, and alcohol and drug use problems among injury survivors with and without traumatic brain injuries. |
